# Making Use of Averaging Methods in MODELLER for Protein Structure Prediction

**DOI:** 10.3390/ijms25031731

**Published:** 2024-01-31

**Authors:** Serena Rosignoli, Elisa Lustrino, Iris Di Silverio, Alessandro Paiardini

**Affiliations:** Department of Biochemical Sciences, Sapienza University of Rome, 00185 Rome, Italy; serena.rosignoli@uniroma1.it (S.R.); lustrino.1856095@studenti.uniroma1.it (E.L.); disilverio.1891816@studenti.uniroma1.it (I.D.S.)

**Keywords:** MODELLER, averaging, lDDT score

## Abstract

Recent advances in protein structure prediction, driven by AlphaFold 2 and machine learning, demonstrate proficiency in static structures but encounter challenges in capturing essential dynamic features crucial for understanding biological function. In this context, homology-based modeling emerges as a cost-effective and computationally efficient alternative. The MODELLER (version 10.5, accessed on 30 November 2023) algorithm can be harnessed for this purpose since it computes intermediate models during simulated annealing, enabling the exploration of attainable configurational states and energies while minimizing its objective function. There have been a few attempts to date to improve the models generated by its algorithm, and in particular, there is no literature regarding the implementation of an averaging procedure involving the intermediate models in the MODELLER algorithm. In this study, we examined MODELLER’s output using 225 target-template pairs, extracting the best representatives of intermediate models. Applying an averaging procedure to the selected intermediate structures based on statistical potentials, we aimed to determine: (1) whether averaging improves the quality of structural models during the building phase; (2) if ranking by statistical potentials reliably selects the best models, leading to improved final model quality; (3) whether using a single template versus multiple templates affects the averaging approach; (4) whether the “ensemble” nature of the MODELLER building phase can be harnessed to capture low-energy conformations in holo structures modeling. Our findings indicate that while improvements typically fall short of a few decimal points in the model evaluation metric, a notable fraction of configurations exhibit slightly higher similarity to the native structure than MODELLER’s proposed final model. The averaging-building procedure proves particularly beneficial in (1) regions of low sequence identity between the target and template(s), the most challenging aspect of homology modeling; (2) holo protein conformations generation, an area in which MODELLER and related tools usually fall short of the expected performance.

## 1. Introduction

In recent years, the field of protein structure prediction has undergone a transformative shift with the introduction of cutting-edge methodologies such as AlphaFold and machine-learning-based approaches [1]. Despite the remarkable accuracy of AlphaFold in predicting static protein structures, it tends to provide predominantly single-conformation representations. This limitation is notable when considering the intricate dynamics and conformational diversity inherent in proteins, where the conventional tool of homology-based modeling continues to be relevant [2,3].

Understanding the dynamics and conformational sampling of proteins is essential for bridging the gap between protein structure and biological function. However, experimentally probing these dynamic features poses significant challenges. In this context, computational methods, particularly molecular dynamics simulations, have become indispensable. Despite their utility, it is important to note that the computational costs associated with these simulations are non-trivial.

Acknowledging the necessity of capturing the dynamic nature of proteins, recent endeavors have explored the application of machine-learning-based tools [4,5,6]. Nevertheless, comparative modeling, employing averaging approaches, can strategically emerge as a key player in elucidating protein dynamics. It also holds potential as a user-friendly methodology accessible to a broad audience. The averaging technique [7,8,9,10] entails employing averaging methods during the refinement phases through two primary steps: (1) utilizing an algorithm that slightly adjusts the spatial coordinates of a given model through Monte Carlo (MC) or Molecular Dynamics (MD) simulations, thus generating many structural replicates of the same sequence, which exhibit both energetic and spatial similarity; (2) collecting a subset of those copies according to an arbitrary criterion and making an average over the atomic coordinates of the selected structures to obtain a refined theoretical prediction of the native structure of the protein.

One of the first assessments validating the improvements associated with averaging were the results brought by Feig and co-workers in CASP10 [7,8]. The averaging basically overcomes the idea of selecting a single snapshot from a generated structural ensemble via a certain energetic criterion, suggesting instead the computation of an ensemble mean. The structures involved in the averaging procedure are chosen along the simulation run consistently with a rank, which is based on energetic evaluations. If, indeed, it is not always true that the structure scoring the lowest energy in a computer simulation corresponds to the one most resembling the native structure, the energy or pseudo-energy functions used are generally trained to recognize a native structure in a set of decoys [8]. Sampling an extensive array of structures during the simulation run is often not crucial to observe improvements resulting from the application of averaging [7,8]. Although not rigorously demonstrated, the enhancements brought about by averaging can be elucidated in two distinct ways. Firstly, the trajectory of the simulation, which is sampled to extract structures for averaging, can be conceptualized as a diffusive process in a high-dimensional space. In this context, the trajectories are predominantly constrained by two free-energy trends. The first trend, affecting residues initially distant from the native structure and from the free energy minimum, corresponds to an essentially flat free energy landscape. Averaging helps mitigate random changes to the starting structure by smoothing out fluctuations. This is especially beneficial as it prevents non-reinforcing alterations. The second trend, influencing residues closer to the native structure and to the free energy minimum, can be assumed to exhibit a harmonic shape. Averaging mitigates random changes to the initial model, effectively refining the position of the harmonic minimum in regions that demonstrate greater conservation throughout the simulation and are consequently closer to the native structure. Secondly, the series of copied configurations of identical structures can be envisioned as the conformation that several identical sequences might adopt when assembled into a crystal. Consequently, ensemble averaging more accurately emulates the results achievable through, for instance, an X-ray crystallography experiment. Notably, a significant finding arises when comparing the results of averaging with the native structure obtained through an NMR experiment. This discovery underscores that the success of the refinement is not confined solely to comparisons with X-ray-determined structures [9,10].

**Figure 1:**
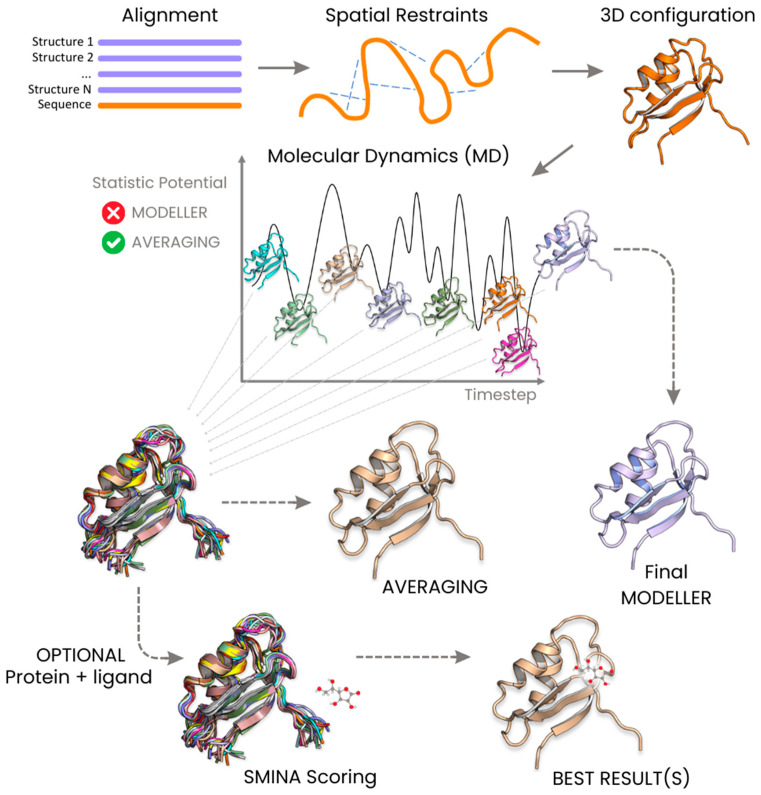
Comparative illustration depicting the workflow of the averaging method in contrast to MODELLER for dynamic protein structure prediction.

Here, we present a novel application of the averaging technique (Figure 1), which is applied during the dynamic path of model building, instead of being limited in its usage to the post-processing phase alone. We tested the results of this kind of approach on the homology modeling software MODELLER (version 10.5, accessed on 30 November 2023) [11]. The MODELLER algorithm utilizes one or more homologous sequences, whose structures are known, as templates for model building. The atoms of the sequence under examination are initially arranged on the topologically equivalent atoms of the homologous templates. Subsequently, a restrained molecular dynamics protocol incorporating a simulated annealing procedure is implemented. This allows for the exploration of the available conformational space for the unknown structure, based on the model restrained by the backbone. The output from MODELLER aligns with an energy-optimized structure generated in the final step of the simulation.

In this study, we leveraged the intermediate structures generated during the MODELLER simulations to construct models through averaging. The proposed algorithm was tested on a meticulously chosen set of 225 target-template pairs to assess whether this averaging-based model construction procedure could improve the overall quality of the generated models.

## 2. Results

### 2.1. Comparing Single Template AVG Models with MODELLER Results

**Figure 2:**
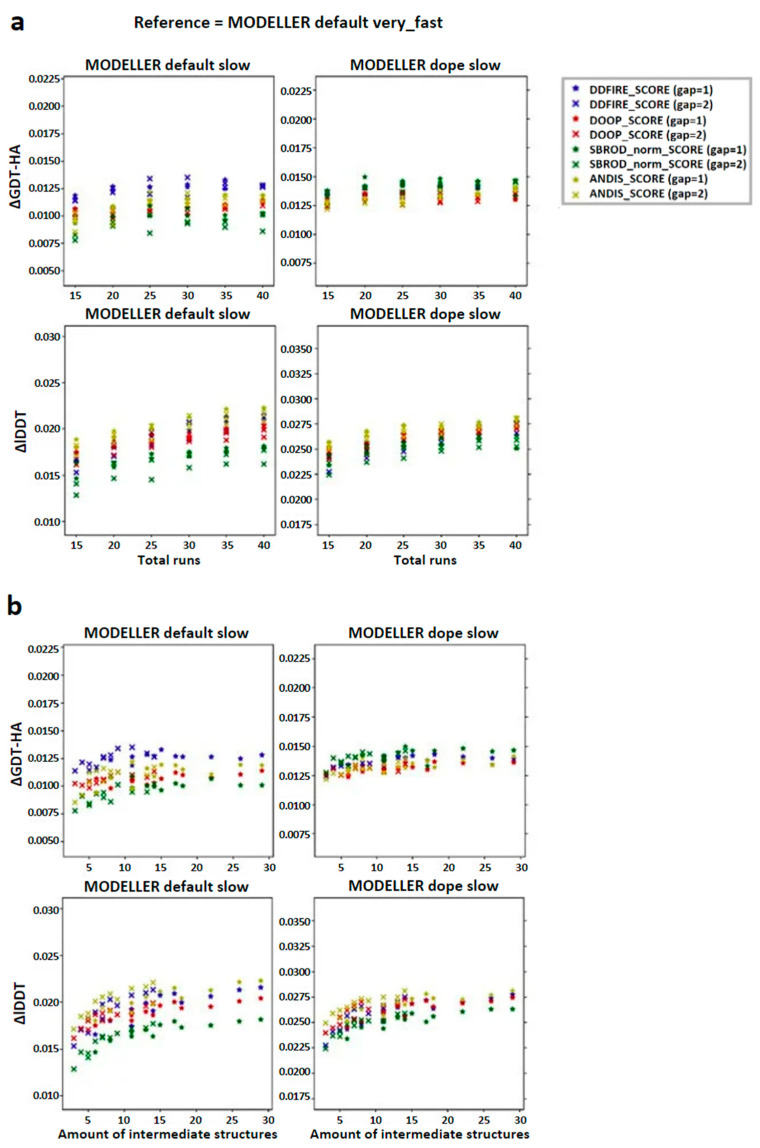
Trend of the global mean differences between the score corresponding to the AVG models and the mean score obtained from the output models of MODELLER when using its original molpdf (default) and the MD protocol, as a function of the number of (**a**) runs used to build the configurations pool and (**b**) intermediate models used to build the AVG-models. The labels “gap” in brackets identify whether the whole pool of configurations was used (gap = 1) or only half of it (gap = 2). The labels ΔGDT-HA and ΔlDDT represent the global mean values of the difference *AVG* − *FINAL* for the given ranking tool. The data refer to the single-template models.

In order to check the existence of a correlation between the number of runs used to build the configurations pool and the quality of the final model, we computed the global mean of the difference between the averaging-built models (AVG-models) and the mean of the MODELLER default very_fast outputs (FINAL-models) with respect to a given assessment score (Local Distance Difference Test (lDDT) and High-Accuracy Global Distance Test (GDT-HA)) and reported the results as a function of the number of runs. The graphs in Figure 2a show that the overall effect of applying the averaging was that of slightly improving the model of the target structure. Another feature that is recognizable from these graphs is that the diversity among the results obtained by different ranking was greater when the structures were extracted from the MODELLER default slow strategy compared to the MODELLER dope slow strategy. This implies that the choice of the statistical potential for ranking has a higher relevance when using the MODELLER default slow configurations pool and that the dDFIRE score, among the tested statistical potentials, might be the most efficient in this case. Moreover, the lDDT graphs show an almost monotonic trend which ends in a plateau, showing that the effect on the lDDT score of the AVG-model was stable when using 30, 35 or 40 runs, while the GDT-HA related graphs show a more homogeneous distribution of the data, especially when the structures are extracted from the MODELLER dope slow strategy, showing that the effect of the number of runs used for the creation of the AVG-models did not affect their GDT-HA score significantly.

The graphs in Figure 2b show, on the other hand, that the quality of the AVG-model might depend more on the number of intermediate models used to build it, rather than the number of runs used to create the configuration pool. Once again, this effect is more evidenced in the graphs concerning the lDDT score, which suggest that at least an amount of 10 structures is needed to get a mean improvement of 0.025 lDDT points, while from the GDT-HA graphs, it is not possible to obtain this information clearly.

**Figure 3:**
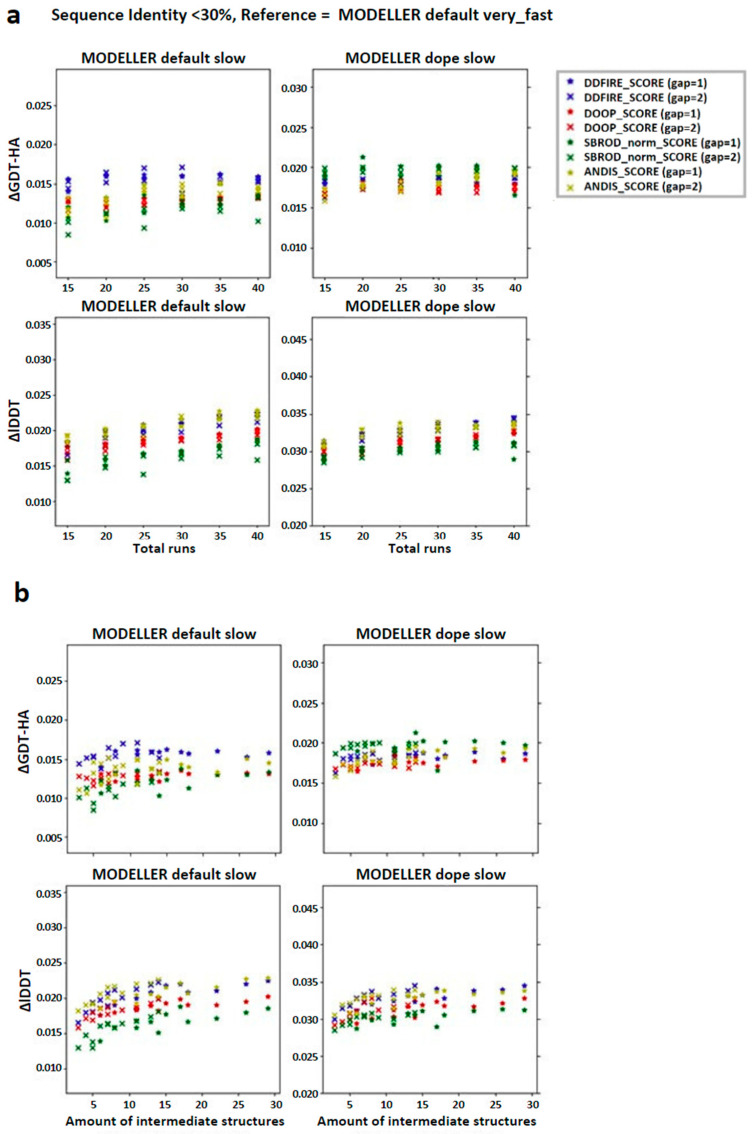
Graphs reporting the trend of the global mean differences, limited to the low sequence identity set, between the score corresponding to the AVG models and the mean score obtained from the output models of MODELLER when using its original molpdf (default) and the MD protocol as a function of the number of (**a**) runs used to build the configurations pool and (**b**) the number of intermediate models used to build the AVGmodels. The labels “gap” in brackets identify whether the whole pool of configurations was used (gap = 1) or only half of it (gap = 2). The labels ΔGDT-HA and ΔlDDT represent the global mean values of the difference *AVG* − *FINAL* for the given ranking tool. The data refer to the single-template models.

**Figure 4:**
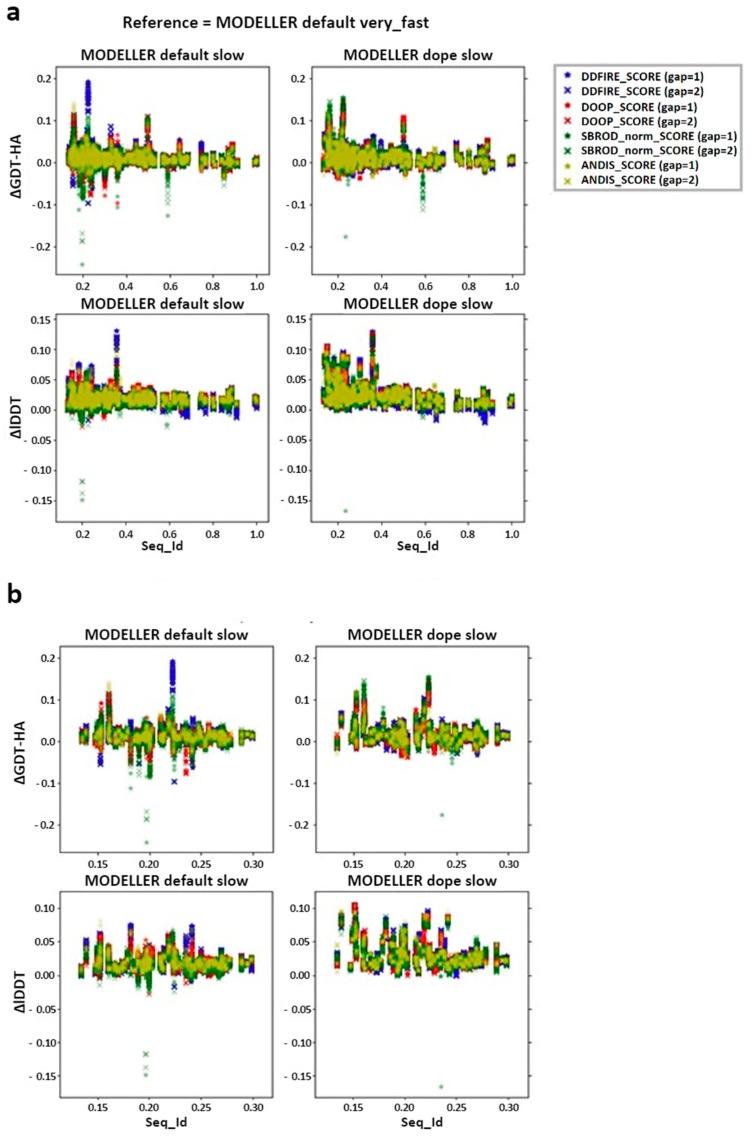
Differences between the score corresponding to the AVG models and the mean score obtained from the output models of MODELLER when using its original molpdf (default) and the MD protocol as a function of the sequence identity between the target and the template structures. (**a**) Whole set (**b**) focusing on the low identity set. The labels “gap” in brackets identify whether the whole pool of configurations was used (gap = 1) or only half of it (gap = 2). The labels ΔGDT-HA and ΔlDDT represent the values of the difference *AVG* − *FINAL* for each pair for the given ranking tool. The data refer to the single-template models.

We then checked the effect of the averaging method on the target-template pairs characterized by low sequence identities (<30%). The graphs shown in Figure 3 suggest that the greater contribution to the mean values of both lDDT and GDT-HA are given by the target-template pairs whose sequence identity was less than 30%. Such observation was also confirmed by the graphs shown in Figure 4a, which report the overall scatter plot of the ΔlDDT and ΔGDT-HA for each target-template pair. This outcome might have been expected, as the homology modeling is a quite efficient tool when given target-template pairs which can be considered as homologous (sequence identity >30%) [5]. This means that the averaging algorithm does not excessively affect the outcome of the model with respect to the one obtained by homology modeling alone.

According to the outcomes reported in Figure 3, the choice of the statistical potential seems to a have a very low influence on the global mean differences, but at the same time, the structures extracted from the MODELLER-DOPE strategy seem to give generally better results, suggesting that the intermediate structures generated during the MODELLER-DOPE runs might score better than those generated by the MODELLER algorithm alone. Focusing the ΔlDDT and ΔGDT-HA per target-template pair to the low sequence identity cases (SeqId < 30%), as shown in Figure 4b, it seems that there was not a defined trend with respect to low sequence identity values, as the data seem homogeneously sparse around a given value above 0.0, especially if we consider the MODELLER-DOPE runs.

### 2.2. Comparing Multiple-Template AVG Models with MODELLER Results

**Figure 5:**
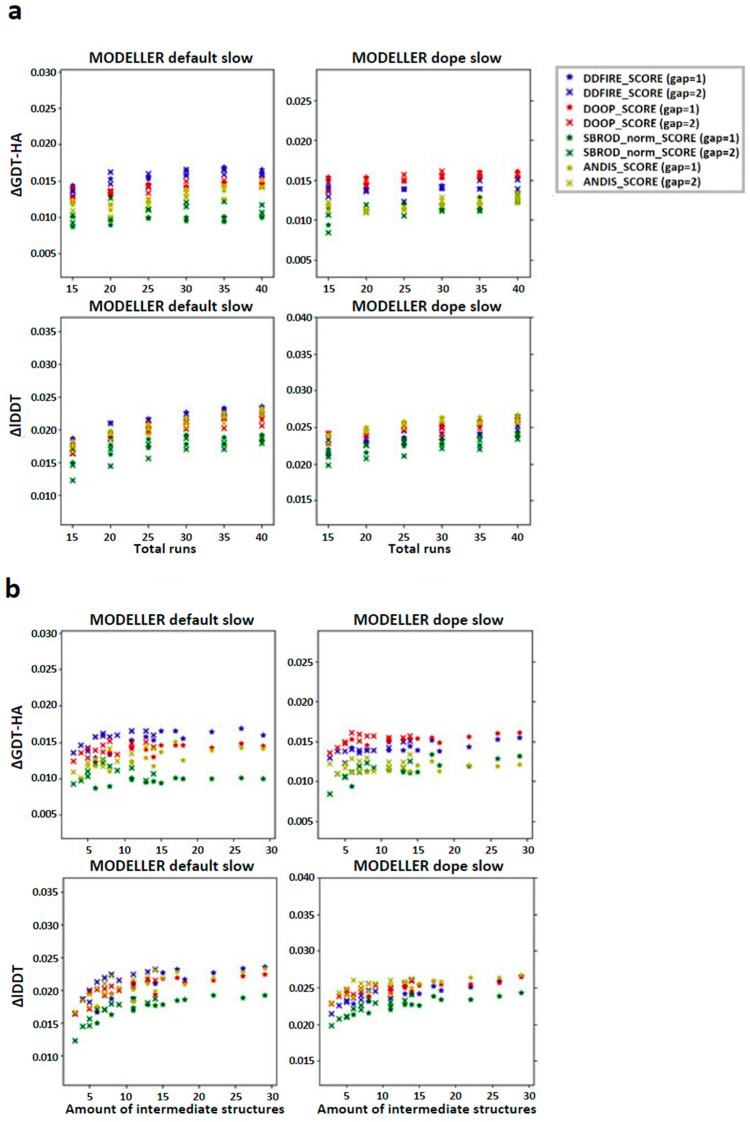
Trend of the global mean differences between the score corresponding to the AVG models and the mean score obtained from the output models of MODELLER when using its original molpdf (default) and the MD protocol, as a function of the number of (**a**) runs used to build the configurations pool and (**b**) intermediate models used to build the AVG-models. The labels “gap” in brackets identify whether the whole pool of configurations was used (gap = 1) or only half of it (gap = 2). The labels ΔGDT-HA and ΔlDDT represent the global mean values of the difference *AVG* − *FINAL* for the given ranking tool. The data refer to the multiple-template models.

The multiple-template set is a collection of 120 targets from the original Analysis Set (AS), which were provided with a number from two to five templates to build the model with [12]. As in the case of the single-template models, the results of the averaging on the multiple-template build models only brought a slight enhancement to the final structure with respect to the MODELLER’s algorithm, and the different ranking methods were all quite equivalent (Figure 5). An ascending trend was obtained for the lDDT evaluations as the number of runs used increased (Figure 5a), and the plateau trending according to the number of intermediate structures used was also found (Figure 5b).

**Figure 6:**
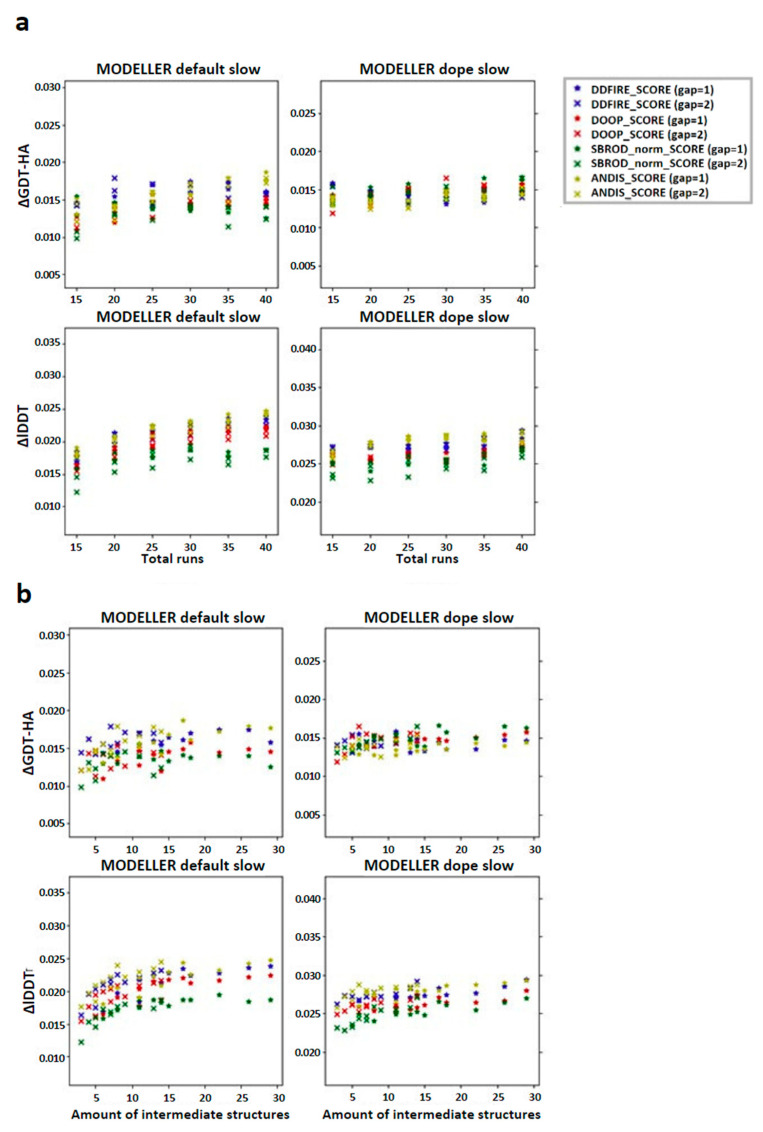
Graphs reporting the trend of the global mean differences, limited to the low sequence identity set, between the score corresponding to the AVG models and the mean score obtained from the output models of MODELLER when using its original molpdf (default) and the MD protocol as a function of the number of (**a**) runs used to build the configurations pool and (**b**) the number of intermediate models used to build the AVGmodels. The labels “gap” in brackets identify whether the whole pool of configurations was used (gap = 1) or only half of it (gap = 2). The labels ΔGDT-HA and ΔlDDT represent the global mean values of the difference *AVG* − *FINAL* for the given ranking tool. The data refer to the multiple-template models.

However, when the AS was reduced to those targets sharing less than 30% of sequence identity with their templates, the increasing trend with respect to the number of runs used was observed only in the lDDT graph concerning the models obtained using the MODELLER default slow intermediate structures (Figure 6a), and even the plateau trend was lost in every case, except for the MODELLER default slow strategy (Figure 6b).

**Figure 7:**
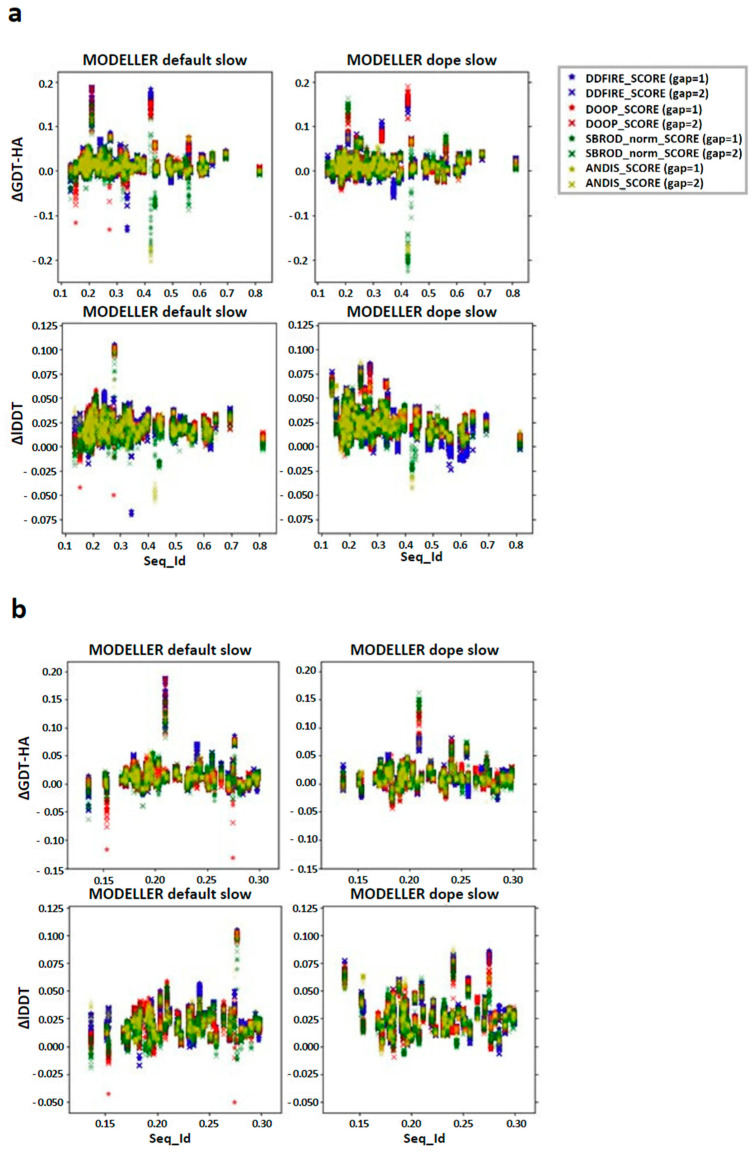
Differences between the score corresponding to the AVG models and the mean score obtained from the output models of MODELLER when using its original molpdf (default) and the MD protocol as a function of the sequence identity between the target and the template structures. (**a**) Whole set, (**b**) focusing on the low identity set. The labels “gap” in brackets identify whether the whole pool of configurations was used (gap = 1) or only half of it (gap = 2). The labels ΔGDT-HA and ΔlDDT represent the values of the difference *AVG* − *FINAL* for each pair for the given ranking tool. The data refer to the multiple-template models.

Regarding the distribution of the differences between the metric calculated on the AVG model and the mean of the FINAL models obtained from the MODELLER strategy, in the case of the multiple templates, there was not an ascending trend toward the lower sequence identities as there was for the single template (Figure 7a); also, in this case, zooming in the low sequence identity region showed a homogeneous scatter plot around a value close to zero (Figure 7b). It is known in the literature that, especially in case of low sequence identity between the target and template, the multiple template procedure is generally enough to enhance the result of a homology modeling prediction [11]. The averaging technique was less efficient on such models, as in every case, the results obtained showed minor values with respect to their single-template counterpart.

### 2.3. Assessing the Ability of the AVG Strategy to Predict Holo Protein Conformations

**Figure 8:**
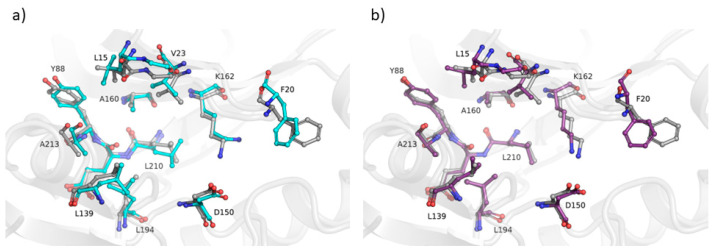
Obtained models of the active site of human Aurora-A kinase with (**a**) default MODELLER pipeline and (**b**) the averaging method. The comparison with the corresponding experimentally determined crystal structure (PDB: 5OSD; grey cartoons and balls-and-sticks) is shown. The residues included in the ATP binding cleft (<4.0 Å from any ATP atom) are shown and highlighted with the corresponding one-letter code and number. Oxygen and nitrogen atoms are colored in red and blue, respectively. Carbon atoms for models in (**a**,**b**) are shown in cyan and violet, respectively.

The “ensemble” nature of the MODELLER building phase can be harnessed, in theory, also to capture low-energy conformations in holo structures modeling (i.e., including heteroatoms in the final model, based on the ligand(s) found in the template structures). Indeed, recognizing the significance of capturing conformational diversity, various pipelines have been developed to address this aspect [13,14]. However, none incorporate protein-ligand scoring, a feature we consider to be essential for enhancing the assessment of averaged models. Since the residue topology and parameter libraries to be used for ligands are usually missing in MODELLER, a “block” residue (BLK) is used to read HETATM records from holo structure templates. These BLKs are treated differently from the other atoms during preparation of dynamic restraints: they are restrained, more or less, as rigid bodies to the conformation of the equivalent residue(s) in the template(s). No chemical information is used. Therefore, we implemented in our pipeline the possibility to make use of the Smina [15] scoring function to identify those low-energy conformations in which the binding site sidechains are optimally positioned to interact with the ligand(s) modeled into the protein structure. Overall, 11 holo structures were modeled by the AVG and MODELLER methods, using as templates the corresponding apo protein and an homologous holo structure bound to the target ligand. Binding pockets were defined to include the residues with at least an atom within 4.0 Å of the ligand found in the experimentally determined structure. We computed all-atom binding pocket RMSDs between each modeled structure and all experimentally determined structures of the same protein. A preliminary comparative analysis of the results (Table 1 and Figure 8) suggests that the AVG approach yields better results compared to MODELLER (mean RMSD was 1.2 and 0.8 for MODELLER and AVG, respectively), being able to capture the ensemble of holo conformations of the protein in which the side chains of the residues interacting with the ligand are well oriented to optimize their contacts with the ligand.

## 3. Discussion

As previously explained, the accuracy of the models produced by homology modeling can be estimated according to the degree of sequence identity between the target and the template. The presented data hint that the overall quality of the MODELLER_dope AVG models slightly overcome the MODELLER_default FINAL and the MODELLER_default AVG models, mostly in the low sequence identity region, while the performance of the MODELLER_dope AVG models is more uncertain in the highest sequence identity region. This latter result is due to the fact that, in these cases, the restraints applied by the MODELLER algorithm result to be very strict, so that the optimized structures picked from the optimized configurations pool are not very likely to differ from the FINAL model regardless of the strategy, lacking so the configurational variety to, at least, move away from the default proposed model. Therefore, the improvement results are confirmed to be greater when the averaging is coupled with the use of the MODELLER_dope strategies [12], and it is, in turn, less dependent on the statistical potential that is applied.

**Figure 9:**
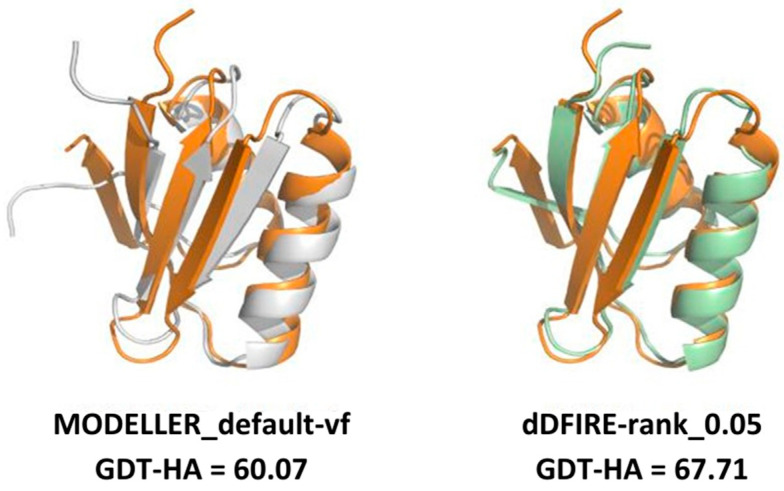
Example of three-dimensional modeling improvement brought by the averaging-building procedure over the default protocol of MODELLER (MODELLER_default). The experimentally determined structure of target_2 (PDB: 1CC8, colored in orange) is superposed to the model with the lowest objective function value from the MODELLER_default strategy (shown in the left image, colored in white) and the dDFIRE-rank model (shown in the right image, colored in pale green). As shown by the GDT-HA values reported below the two images, the averaging-built model displays a higher structural similarity with the target. Template used: 3CJK, % identity 21.88%, coverage: 88.89%.

In this work, the trends observed for the GDT-HA, when taken as a function of the sequence identity between a target and its template(s), are reported. The ΔGDT-HA rarely assumes negative values, especially in the low sequence identity region (Figure 4 and Figure 7). Such trend indicates that the combination of MODELLER_dope simulations together with averaging procedures have a moderate efficiency in improving the quality of the models, even when the optimized averaging set is constructed by picking randomly from the configurational pool. However, it is evident from the presented data that the extent of such improvement is strongly dependent on the metric (GDT-HA vs. lDDT) and on the statistical potential used for ranking. In a best-case scenario, the averaging procedure brought a net gain in the performance of the model building, as reported for example in Figure 9. However, in general, our results show that, though averaging technique is potentially a powerful tool in the refinement of protein structure modeling, its effects in the building phase of the model do not seem to be outstanding, as it would be expected. Moreover, the computational power needed to use the averaging algorithm overcomes the slight improvement of the quality of the results which are obtained by the technique. Nevertheless, although the improvements obtained do not usually overcome the threshold of a few percentage points in the evaluation of the mean GDT-HA, the results are nonetheless relevant, since such a grade of difference is found to be relevant at CASP competition level. For example, it must be noted that a ∼3.5% improvement in the mean GDT-HA corresponds to the difference in TBM performance between the first server in CASP 12 and the server at position five [17]. Furthermore, the most valuable result is that it was seen that the effect of the averaging-building procedure generates enhancements especially in the region of low sequence identity between the target and the template(s). In addition, the implementation of external potentials in the objective function of MODELLER definitely biases the outcome toward a better similarity with the native structures. As shown before, this is particularly true when implementing the Smina scoring function to identify low-energy holo conformations of ligand-bound models. Indeed, since protein binding clefts are highly flexible, each protein can adopt multiple conformations that depend whether a ligand is present, and which one is bound. We thus evaluated the accuracy of each model generated during the AVG phase using the Smina scoring function and by computing the binding cleft RMSD to that of the corresponding available experimental structure of the same protein. We then compared the obtained results to the MODELLER procedure, and we showed that holo conformations are more accurately described by the highest-ranking models extracted by the AVG method, when optionally implemented with the Smina scoring function, than to the best models obtained by MODELLER. These results have important implications for protein structure prediction in drug discovery, especially if we consider that, until now, AlphaFold and similar AI-based tools do not allow one to specify a bound ligand when generating a protein model, and most importantly, the accuracy of ligand binding poses predicted by computational docking to AlphaFold models is much lower than when docking to experimentally determined apo proteins [18].

## 4. Materials and Methods

### 4.1. Statistical Potentials

We used a statistical potential-based ranking as a criterion to apply the averaging phase on the intermediate structures of the simulation [19,20]. Statistical potentials are pseudo-energetic functions designed with two main aims: being able to recognize a native structure in a set of decoys and identify the most accurate model for a target structure in a set of given three-dimensional models. These mathematical tools are based on potentials of mean force and exploit the properties of the Boltzmann distribution to estimate the strength of a given interaction according to the frequency it is measured within a protein set. This method aims to estimate these probabilities in a general manner, so that the most significant interactions in the building of the three-dimensional structure of a protein is highlighted. In this work, we tested dDFIRE [21], SBROD [22], DOOP [23], and ANDIS [24] as ranking criterions to select the intermediate configurations which would be used to create our averaging-built model.

### 4.2. MODELLER Algorithm and Its Variants

MODELLER is a comparative modeling software developed by Andrej Šali in 1989, and it is one of the most used tools in model building, due to its accuracy and low computational requirements [11]. The computational part is executed by a collection of algorithms written in Fortran code, but MODELLER is implemented as an interpreter of a high-level language specialized for dealing with protein structures. The version used in this work is MODELLER 9.21, which uses the CHARMM22 force field in the execution of the simulation runs. In MODELLER, the comparative modeling algorithm is based on the satisfaction of spatial restraints method. Given an alignment of the sequence to be modeled (the target) with the related protein structures (the templates), the satisfaction of spatial restraints happens in two steps: (1) extraction of spatial restraints using the information derived from the alignment; (2) satisfaction of the restraints to obtain a 3D model.

The restraints in MODELLER are expressed as probability density functions (which will be referred as pdfs) associated with the geometric and structural features of the protein, such as distances between main-chain atoms, dihedral angles, distances between atoms belonging to different residues and so on. Using only the pdfs associated with individual features (represented as $p_{F}\left(f_{i}\right)$) is insufficient for running a simulation that accurately captures the native structure of the target protein (see Equation (1)):

(1) $$P=\prod_{i}\;p_{F}\left(f_{i}\right).$$

To address this limitation, pdfs were assumed to be independent of each other and were combined through a product to derive the molecular pdf associated to the protein structure (which is referred to as molpdf):

(2) $$p_{F}\left(f_{i}\right)=p_{Phy}\left(f_{i}\right)p_{Hom}\left(f_{i}\right).$$

The term $p_{F}\left(f_{i}\right)$ is expressed as the joint probability of the physical ($p_{Phy}\left(f_{i}\right)$) and homology ($p_{Hom}\left(f_{i}\right)$) (see Equation (2)) restraints associated with the feature, which are assumed to be independent as well. The purpose of a definition of a molpdf is due to the necessity of having a function to be optimized along the simulation to obtain the most probable configuration for a sequence of unknown structure. Due to errors associated with floating points, the function which is optimized during the simulation is actually a transformation of the molpdf (see Equation (3)), which is called the objective function. This transformation turns the molpdf in an additive quantity, turning the productory contained in P into a sum over the transformations of the feature pdfs:

(3) $$F=-ln\left(P\right)=-\sum_{i}\;l\left(p_{F}\left(f_{i}\right)\right)\;.$$

The objective function can be expressed as the sum of a physical objective function $F_{Phy}$ and a homology objective function $F_{Hom}$ (see Equation (4)), since the form of the single $p_{F}\left(f_{i}\right)$ is the product of $p_{Phy}\left(f_{i}\right)$ and $p_{Hom}\left(f_{i}\right)$ (see Equation (2)). The term $F_{Phy}$ considers only the CHARMM22 contributions (covalent bond length potential, bond angle potential, stereochemical cosine torsion potential, stereochemical improper torsion potential), while the $F_{Hom}$ is composed by a combination of harmonic terms (associated to the Gaussian definition of the $p_{Hom}\left(f_{i}\right)$, see [11]) dealing with the interatomic distances of the model (Carbon α distance restraints, main-chain nitrogen and oxygen distance restraints, side-chain–main-chain distance restraints, side-chain–side-chain distance restraints), whose equilibrium positions are set around the corresponding values of the templates [12].

(4) $$F=F_{Phy}+F_{Hom}.$$

The aim of the algorithm therefore becomes the minimization of the objective function F. Since the objective function represents the free energy associated to the three-dimensional configuration of the protein, given its additive property, it is possible to further add energetic terms in order to try to achieve a more accurate energy minimization, since most of the time, the $F_{Hom}$ term drives the final model in a structure which is too close to the template structure, and the $F_{Phy}$ term is not sophisticated enough to drive the final model neither in a structure closer to the target, nor deal with the random walk characterizing disordered or loop regions of the structure. In [12], an attempt was made to customize the objective function by adding the DOPE score terms [25], resulting in an improvement of the final model of the structure. In this work, we therefore tested our averaging method by applying it on sets of configurations extracted both from simulations run with the default molpdf and the DOPE-molpdf.

Additionally, MODELLER provides five MD routines, which differ by the amount of timesteps composing them (for more information, visit https://salilab.org/modeller, accessed on 30 November 2023). In this work, we used the “slow” routine, which is suggested in order to have a reasonably correct model for the target structure, due to the wider configurational space explored, compared to the default mode “very fast”.

### 4.3. Analysis Set

The Analysis Set (AS) includes 225 target-template protein structure pairs, experimentally resolved by crystallography, and the exact procedure according to which the AS was created is reported in [12]. It was constructed from Protein Data Bank (PDB) collected structures exploiting the software PISCES (https://pisces.ucdavis.edu/, accessed on 30 November 2023), which filters the PDB and extracts sets of structures with specific features; the high number of structures contained in the PDB makes it, indeed, extremely redundant, as many structural files correspond to the measure of one sequence structure in different experimental conditions. In the obtained AS, the target structures contained are not homologous, sharing less than 10% of amino acid sequence with each other, and the template sequence identity with the corresponding target ranges from 10% to 99%. The target-template alignments in this set were built using the HHalign sequence alignment program. The construction method of the AS provides a protein set representative of the information inferable from the non-redundant structures collected in the PDB. Thanks to the heterogeneity of the AS, it is possible to carry out an analysis on the general performance achieved by the modifications produced with respect to the default MODELLER algorithm. In order to do so, in the analysis, we report quantities referred as the global-mean, whose nomenclature indicates that, given a feature A calculated on a single target-template analysis (for example, the result of the structural assessment of one model), its global-mean corresponds to the arithmetic mean of the features A computed singularly on the N targets of the AS, as shown in Equation (5):

(5) $$A_{global\_mean}=\frac{1}{N}\sum_{i=1}^{N}\;A_{i},$$

where $A_{i}$ represents the value of the feature A calculated for the *i*-th target. 

The global-mean will be exploited to gain an overall comparison of the produced models, mainly taking the ones produced by the default MODELLER algorithm as reference. The statistical relevance of the results obtained from such comparisons was assessed by the Wilcoxon signed-rank test. The Wilcoxon signed-rank test is a non-parametric, statistical-hypothesis test commonly used to compare aspects characterizing two related sets by computing and ranking the differences between them. The null hypothesis of this test is that the results calculated on the two compared sets are distributed according to the same probability distribution, whose knowledge is not fundamental to carry out the test. The probability-value (*p*-value) outcomes of the Wilcoxon test assess whether the differences obtained are due to statistical fluctuations or emerge from an underlying distinction in the feature distributions governing the two different sets. The statistical relevance of the eventual changes in the global-mean due to the averaging procedure was evaluated by calculating the Wilcoxon test *p*-values, *p*-value = 5% as the confidence threshold.

### 4.4. Building a Configurations Pool

The basis to gain a good performance from an averaging algorithm is the possibility to have the availability of a broad sample of configurations corresponding to the same structure, from which a certain number of models to use in the averaging procedure is derived [9]. Such a sample will be further referred to as “configurations pool”. In this work, the averaging-building algorithm was tested on two different MODELLER strategies, which differ in the definition of the objective function (default molpdf and the DOPE-molpdf, as explained in “MODELLER algorithm and its variants”). Since the sample of intermediate structures extracted from one MODELLER run alone did not provide a sufficiently large configurations pool, we tested our algorithm by creating six configurations pools, merging the intermediate steps from 40, 35, 30, 25, 20, and 15 MODELLER runs for each target-template pair from the AS, in order to obtain a hint on the minimum number of runs requested to obtain suitable results. Each run differed only by the initial, randomly perturbed configuration (https://salilab.org/modeller, accessed on 30 November 2023), and the merging operation was also legitimated by the reported results that the parallel production of multiple samples usually leads to a production of better averaging refined models [9]. Due to the small value assigned to the timestep in MODELLER (4ps), the dynamics followed by the structures should not be considered as an actual representation of physico-chemical phenomena, while it should be rather seen as a means to perturb the structure and explore a limited area in a configurational space. Therefore, the intermediate frames which are created within consecutive timesteps in a MODELLER simulation usually show a strong correlation, generating an effect of redundancy of models in the total intermediate sample. According to this outcome, we assumed that collecting the intermediate structures at each step of the simulation would not bring any significant improvement to the averaging procedure; this justifies the reduction applied on the amount of data to collect, which would have been uselessly too computational expensive both to save and analyze. The number of extracted structures was adjusted by empirically evaluating the differences between two consecutive models produced by the very_fast procedure; then, the number of frames extracted from the slow procedure was chosen in order to have a set of intermediate models comparable with the set obtained by the very_fast procedure. The two MODELLER strategies reported were performed using the slow molecular dynamics routine. Given a MODELLER strategy, the intermediate non-optimized models (called raw models) associated with each run were extracted every 34 timesteps and stored, resulting in the collection of 149 intermediate structures from each simulation, regardless of the objective function which characterizes the strategy. We also tested the algorithm, taking only half of the pool of configurations, by excluding the structures corresponding to odd timesteps.

We tested the algorithm by extracting the 0.3% and 0.5% top-ranking structures from the configurations pool ensembles; such structures were exploited to generate the averaging-built models. Finally, the averaging-built models underwent a conjugate gradient optimization, which is needed to avoid possible stereochemical inconsistencies of the final model. The objective function used in the conjugate gradient optimization corresponds with the objective function of the DOPE-molpdf MODELLER strategy.

### 4.5. GDT-HA and lDDT Metrics

We employed the High-Accuracy Global Distance Test (GDT-HA) [26] and the Local Distance Difference Test (lDDT) [27] scores as criteria for assessing the reliability of the obtained models with respect to the corresponding experimentally known structures.

The Local-Global Alignment (LGA) method facilitates the comparison of protein structures or specific fragments of protein structures by creating many different local superpositions to identify regions of similarity between proteins. The LGA scoring function has two components: the Longest Continuous Segments (LCS), for the detection of local regions of similarity by focusing on the RMSD, and the Global Distance Test (GDT), also named as the Total Score GDT (GDT-TS), designed to find regions of global structure similarity by focusing on distance. The High-Accuracy GDT (GDT-HA) score [28] is the high–precision version of the GDT-TS score, featuring smaller distance cutoffs (distance cutoffs of 1, 2, 4, and 8 Å are replaced with distance cutoffs of 0.5, 1.0, 2.0, and 4.0 Å) [29].

The Local Distance Difference Test (lDDT) is a superposition-free score used to assess protein structure models in comparison to a reference structure. This evaluation is achieved by measuring the local distance differences among all atoms within a model. The lDDT score offers a metric to gauge how well the environment of a reference structure is replicated in a protein model [27].

### 4.6. MODELLER Trajectories Analysis

**Figure 10:**
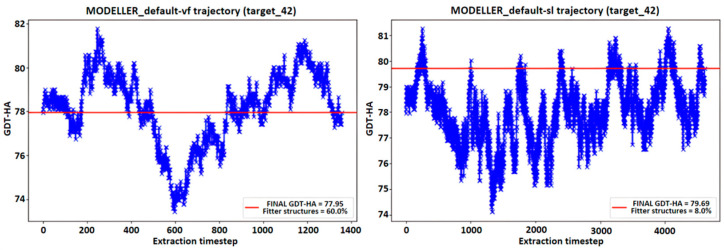
Trend of the GDT-HA metric of the intermediate structures in a MODELLER run, compared to the experimentally determined target structure. The red line represents the GDT-HA value of the final structure of the simulation, and the value “Fitter structures” indicates the percentage of intermediate structures which outperformed such a result.

The idea behind the averaging procedure resides in the hypothesis that, due to the peculiar pseudo-energy landscape explored during a simulation, there is a chance for the existence of intermediate models which are more consistent with the target structure than the ones proposed by default by MODELLER; to the best of our knowledge, such hypothesis has not yet been investigated in literature. An example of the MD’s trajectories representing the GDT-HA calculated on the intermediate models produced in a single MODELLER simulation is reported in Figure 10. This, basically, represents the trend of the accuracy of the generated intermediate models along the simulation. It appears that there are models whose similarity to the target structure results more accurately than the one assessed on the FINAL model. This result is shown in Figure 10 by displaying a horizontal red line representing the value of the GDT-HA [28] metric between the FINAL model of the simulation and the experimentally determined target structure, noticing that a fraction of the intermediate frames from the MODELLER trajectory surpasses the threshold signed by the horizontal line.

### 4.7. Ranking by Means of Statistical Potentials

The selection procedure by ranking was carried out as follows: (1) The intermediate models were ranked according to the scores of one statistical potential, calculated on the raw configurations pool. (2) After ranking, only a fraction of the top structures of the classification was selected, forming the raw averaging set. This set was used to pick the models from the optimized configurations pool, to finally carry out the averaging procedure. In this work, the effect of averaging was tested by selecting the top 0.3% and top 0.5% models of the rank. (3) The models to be used in the averaging procedure were selected from the optimized configurations pool by picking the optimized version of the structures contained in the raw averaging set, composing the optimized averaging set.

We used the GDT-HA [28] and the lDDT [29] scores to compare the models obtained with the respective experimentally known structures, assessing the reliability of the model with respect to the experimental data. We reported the results comparing both the GDT-HA and the lDDT scores obtained with the averaging-built models (hereinafter “AVG-models”) with the mean scores obtained from the output models of MODELLER default strategy (hereinafter “FINAL-models”), which is characterized by the default-molpdf and the very-fast MD, and with the output models built using the strategy from which the configurations constituting the AVG-models were extracted (respectively, default-models and dope-models, overall strategy-models). Such comparison was expressed as an arithmetical difference between the score obtained for the single AVG-model, and the mean score obtained from the output models were used to create the configurations pool.

## 5. Conclusions

This study explores the impact of implementing the averaging technique during the homology modeling building phase, employing the MODELLER algorithm. Intermediate structures extracted from MODELLER runs were ranked based on various scores, and the selected structures were used to build a model through position averaging. The effectiveness of this approach relies on refining ranking scores closely linked to native protein structures. An innovative aspect of the algorithm developed in this project is the integration of a refinement tool within the MODELLER-based model building phase. This eliminates the need for additional simulations beyond homology modeling, simplifying the acquisition of the required pool of configurations. The algorithm is available at https://github.com/pymodproject/Averaging/tree/main, (accessed on 30 November 2023).

## Figures and Tables

**Table 1:** Comparative analysis of the Root Mean Square Deviation (RMSD) values obtained from two different modeling methods: the default MODELLER approach and our proposed averaging method. The table showcases the performance of both methods across a dataset of 11 proteins [16], with the final row presenting the mean RMSD values for each method, illustrating the overall effectiveness and improvement offered by the averaging method in homology modeling.

Protein	PDB Apo	PDB Holo	Holo Ligand	PDB Template	Template Ligand	%id	RMSD Default Modeller	RMSD Averaging Method
Carbonic anhydrase II	2CBE	1BCD	FMS	4YGF	AZM	29.3	0.5	0.2
Peroxisome proliferator-activated receptor γ	1PRG	2GTK	208	6NWS	L77	28.0	1.7	0.5
Vascular endothelial growth factor receptor kinase	1VR2	2P2I	608	6P3D	0LI	29.1	0.8	0.7
Rac-beta serine/threonine protein kinase	1GZK	3D0E	G93	6BXI	ANP	29.6	0.9	0.9
AmpC *β*-Lactamase	2BLS	1L2S	STC	4E6W	APB	26.1	0.9	0.5
Catechol O-methyltransferase	4PYI	3BWM	SAM	5ZW4	SAM	25.2	3.2	0.8
Beta-secretase 1	2ZHV	3L5D	BDV	1PPM	0P1	27.5	2.2	2.0
Fatty acid-binding protein, adipocyte	3RZY	2NNQ	T4B	2IFB	PLM	29.2	0.6	0.3
Peroxisome proliferator-activated receptor δ	2GWX	2ZNP	K55	7KXD	Z7G	27.4	1.5	0.6
Blood coagulation factor Xa	1HCG	3KL6	443	6ZOV	GBS	27.4	0.5	0.3
Aurora kinase A	4J8N	5OSD	ADP	3D5W	ADP	34.1	0.6	0.3
	**Mean RMSD**						1.2	0.8

## Data Availability

Data is contained within the article.
